# Impact of “national suicide prevention week” on digital awareness of suicide prevention : an insight from google trends

**DOI:** 10.1192/j.eurpsy.2021.1082

**Published:** 2021-08-13

**Authors:** C. Trivedi, S. Shukla, M. Adnan, K. Shah, L. Weiss

**Affiliations:** 1 Research, St Davids Healthcare, Austin, United States of America; 2 College Of Natural Science, University of Texas at Austin, Austin, United States of America; 3 Psychiatry, Mercy Hospital and Medical Center, Lincolnwood, United States of America; 4 Department Of Psychiatry, Griffin Memorial Hospital, Norman, United States of America; 5 Psychiatry, Psychiatry Austin, Austin, United States of America

**Keywords:** Mental Health Policy, Suicide, Suicide prevention, Mental Health awareness

## Abstract

**Introduction:**

Every year in the month of September, National Suicide Prevention Week is celebrated. The goal of suicide prevention week is to inform the public about suicide prevention, primarily the warning signs of suicide. However, the impact of this month on the general population is unknown. The Google trends show how frequent web searches have been performed for a particular search-term, which provide an approximation of the people’s interest.

**Objectives:**

To evaluate public interest in suicide prevention by analyzing the google trends of “Suicide Prevention” search-term.

**Methods:**

We estimated the interest in such topics by running the google trends data of the last decade by using the filter [Search Term:“Suicide Prevention”, Locations: “United States” and Time Ranges “ 2010 to 2020”].

**Results:**

During this specific interval, people have searched “Suicide Prevention” most frequently during the month of September (month of National Suicide Prevention week). Conversely, in the other months, interest in “suicide prevention” fluctuated between little to none. The only other time people have shown interest in Suicide prevention, other than the month of September, was with suicide news in the media, such as the death of a celebrity by suicide, or suicide-related TV shows. [Figure]

**Figure:**
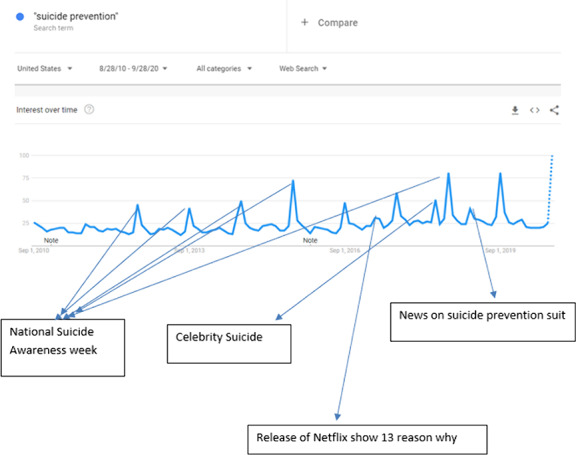

**Conclusions:**

Although it is not definitive, it gives some idea that National Suicide Prevention week has a considerable impact on population interest. Since we did not observe sufficient public interest in other months, there should be frequent and systematic efforts to spread suicide prevention awareness among the general population.

